# Hepatitis Associated Aplastic Anemia: A review

**DOI:** 10.1186/1743-422X-8-87

**Published:** 2011-02-28

**Authors:** Bisma Rauff, Muhammad Idrees, Shahida Amjad Riaz Shah, Sadia Butt, Azeem M Butt, Liaqat Ali, Abrar Hussain, Irshad-ur-Rehman, Muhammad Ali

**Affiliations:** 1 Division of Molecular Biology, National Centre of Excellence in Molecular Biology (CEMB), University of the Punjab, 87 West Canal Bank Road, Thokar Niaz Baig, Lahore, 53700, Pakistan

## Abstract

Hepatitis-associated aplastic anemia (HAAA) is an uncommon but distinct variant of aplastic anemia in which pancytopenia appears two to three months after an acute attack of hepatitis. HAAA occurs most frequently in young male children and is lethal if leave untreated. The etiology of this syndrome is proposed to be attributed to various hepatitis and non hepatitis viruses. Several hepatitis viruses such as HAV, HBV, HCV, HDV, HEV and HGV have been associated with this set of symptoms. Viruses other than the hepatitis viruses such as parvovirus B19, Cytomegalovirus, Epstein bar virus, Transfusion Transmitted virus (TTV) and non-A-E hepatitis virus (unknown viruses) has also been documented to develop the syndrome. Considerable evidences including the clinical features, severe imbalance of the T cell immune system and effective response to immunosuppressive therapy strongly present HAAA as an immune mediated mechanism. However, no association of HAAA has been found with blood transfusions, drugs and toxins. Besides hepatitis and non hepatitis viruses and immunopathogenesis phenomenon as causative agents of the disorder, telomerase mutation, a genetic factor has also been predisposed for the development of aplastic anemia. Diagnosis includes clinical manifestations, blood profiling, viral serological markers testing, immune functioning and bone marrow hypocellularity examination. Patients presenting the features of HAAA have been mostly treated with bone marrow or hematopoietic cell transplantation from HLA matched donor, and if not available then by immunosuppressive therapy. New therapeutic approaches involve the administration of steroids especially the glucocorticoids to augment the immunosuppressive therapy response. Pancytopenia following an episode of acute hepatitis response better to hematopoietic cell transplantation than immunosuppressive therapy.

## Background

Aplastic anemia, acquire or congenital anemia associated with hypoplastic "fatty or empty" bone marrow and global dyshematopoiesis, has been first described by Paul Ehrlich in year 1888 [1]. The pathophysiology is believed to be idiopathic [2] or immune-mediated phenomenon with active destruction of haematopoietic stem cells [3]. The abnormal immune response may be elicited by environmental exposures, such as to chemicals, drugs, viral infections and endogenous antigens generated by genetically altered bone marrow cells [4]. A small fraction of the genes involved in pancytopenia has been represented by the congenital BM failure syndromes (relatively rare) which lately develop in clinical syndromes as Fanconi's anemia, Dyskeratosis Congenita, and Shwachman-Diamond syndrome [5,6].

Hepatitis-associated aplastic anemia (HAAA) is a well recognized and distinct variant of clinical syndrome, acquired aplastic anemia, in which an acute attack of hepatitis leads to the marrow failure and pancytopenia [7-9]. HAAA has been first reported in two cases by Lorenz and Quaiser in 1955 [8] and the number of the cases increase up to value of 200 by the year 1975 [10-12]. However, this syndrome has been reported in 2-5% cases of west and 4-10% in area of more prevalent to hepatitis and Human Immunodeficiency Viruses (HIV) in the Far East [12,13], it belongs to the area of low socioeconomic status [14,15]. HAAA is not considered relative to age, sex and severity of hepatitis [14], predominantly it has been found in children [13], adolescent boys and in young aged men [10,16].

The onset of syndrome, pancytopenia, usually takes two to three months [62 days: ranging from 14 to 225) after attack of acute hepatitis [12,14]. Hepatitis associated with aplastic anemia may be acute and chronic [**7**], mild and transient [16], self-limiting and fulminant and the development of AA is always fatal if not treated on time [7,10]. Majority of the cases have been found as fulminant where the mortality rate reaches up to 85% [17]. Aetiology of the syndrome has been attributed to various agents and factors [**7**] which may include pathogenic viruses, autoimmune responses, liver transplantation procedure [18] bone marrow transplantation, radiation [19] and drugs administered to control the viral replication [14].

## Aplastic anemia associated hepatitis viruses

Several hepatitis viruses such as Hepatitis A [20], B [21,22], C [23], and E, G [24] have been anticipated to be associated with this set of symptoms [7]. No association has been found with blood transfusion, toxins and drugs [17]. In one study Safadi and co-workers (2001) found out that sera of eight of the patients had the existence of Hepatitis Bc IgG and/or anti-HBs antibodies which was suggested due to past exposure and immunizing effect and they were unable to establish any direct relation with acute hepatitis B [14]. As non-A, non-B hepatitis agents are usually responsible for the hepatitis associated aplastic anemia; the prevalence of anti-hepatitis C virus antibodies is similar in HAAA and aplasia of other origins [25]. HCV seropositivity has been observed in the patients developing cytopenia following non-A non-B hepatitis (NANBH). HCV viremia has been frequently observed without detecting anti-HCV antibodies in patients' blood reflecting the transfusion associated HCV infection [25,26]. However, it has also been reported that HCV is not generally implicated as a causative agent of hepatitis preceding aplastic anemia [27].

Hepatitis G virus (HGV) has been reported as a possible etiological agent of acute hepatitis, chronic liver dysfuntioning, and fulminant hepatitis and hepatitis associated aplastic anemia. A relation of Hepatitis G virus with hepatitis subsequently developing in the aplastic anemia has been seen in a 24 years old person by measuring the hematological, biochemical, serological and virological parameters and detecting HGV RNA in his serum by PCR and electro-immunoassay [24].

The development of aplastic anemia generally found to occur in hepatitis not caused by the hepatitis A and hepatitis B viruses commonly known as the non-A and non-B hepatitis associated aplastic anemia which were reported in above than 80% of the cases of hepatitis preceding severe cytopenia [28,29].

## Viruses other than hepatitis associated with aplastic anemia

Viruses other than the hepatitis viruses have also been implicated as a causative agent of AA [1,5] which include parvovirus B19 [19,30,31], Cytomegalovirus, Epstein bar virus [19,31,32], Echovirus 3 [33], GB virus-C [34], Transfusion Transmitted virus (TTV) [35], SEN virus and non-A-E hepatitis virus (unknown viruses) [7].

Parvovirus B19, an under recognized hepatotrophic virus, is documented as an offending agent of HAAA. Its infection cause hepatic manifestation ranging from abnormal liver functioning to Fulminant Hepatic failure and aplastic anemia [30]. The primary site of infection of this virus is erythroid progenitor cell in which it halts the erythropoises and leads to the anemia in immunocompromised hosts. The DNA of this virus has been detected in liver of fulminant hepatic failure manifested with bone marrow aplapsia and in the serum of fulminant hepatitis children of unknown origin [36,37].

Association of Torque Teno virus, single stranded circular DNA has liver as a susceptible host as well as various other tissues including bone marrow. It has been firstly reported in 12 years old Japanese boy suffered from cytopenia following acute hepatitis by detecting Torque Teno virus DNA of genotype 1a and IgM antibodies against this virus in peripheral blood and bone marrow mononuclear cells which precludes that acute bone marrow failure majorly concerns with infection of TTV virus to haemopoietic progenitor cells. However, other studies have also been done on assessing the TTV as an etiological agent of HAAA [33,38].

In a study a 6-year-old boy was experienced aplastic anemia two months after the onset of acute hepatitis associated with echovirus-3 [33]. As idiopathic aplastic anemia is associated with the increased level of secretion of INF-γ and TNF-α from T cells which inhibit the hematopoietic cell proliferation [39], similar increased level of serum INF-γ and TNF-α was found in the patient four weeks after the onset of aplastic anemia [33]. Similarly varying degrees of cytopenia has been related to the HIV infection and severe aplastic anemic conditions develop after subsequent attack of HSV-6 [17,40].

Epstein Bar virus infection, involved in hepatitis, manifests the pathogenesis of marrow aplasia [40]. The pathogenic mechanism involve in the EBV infection is direct cytotoxity or mediates the immune response of host [7,17,14,41].

## Immunopathogenesis of HAAA

Aplastic anemia can be acquired or congenital [1,42]. As the aplastic anemia following the hepatitis has been elucidated as a severe bone marrow failure with an episode of acute hepatitis, following lymphocyte variations occur during the course of the syndrome: activation of circulating cytotoxic T cells increase, tend to accumulate in the liver, broad skewing patter of T cell reportrie in peripheral blood of the patient forms, a large number of T cell infiltration from liver parenchyma occurs [13,26,43,44] defective monocyte to macrophage differentiation [45] and decreased circulating level of interleukin-1 occur [46].

Various Immunological abnormalities have been accountable for the development of aplastic anemia following hepatitis. The immunological abnormalities with HAAA show that CD8+ kupffer cells detecting by liver biopsies appear as a mediator of this syndrome. In a study it has been reported that patient showed a decreased ratio of CD4/CD8 cells and a high percentage of CD8 cells which can be cytotoxic and myleopoietic during the in vitro study of aplastic anemia [10,20]. The residing of CD8 cells in bone marrow during HAAA produces a high level of interferon gamma (INF-γ) and cells derived from bone marrow locating in liver may activate these cytotoxic T cells causing their intrahepatic accumulation strongly affected by the Tumor necrosis factor alpha (TNF-α) and interferon gamma (INF-γ) causing the onlooker damage to liver cell of genetically modified mouse model. However, it has also been shown in several studies that increased level of soluble IL-2 receptor forms the major reason of non specific inflammation of HAAA [47,48]. The pathogenesis of HAAA in children has been suggested to relate with the interruption in balance of lymphocyte sub-populations and T lymphocyte activation [49].

## Genetic Vulnerability of HAAA

Being an acquired disease, severe HAAA has also been presented as a Familial Bone Marrow Failure Syndrome (FBMFS) in a study while finding the family donor of HSCs transplant for treatment of idiopathic fulminant liver failure patient who has developed myelodysplastic syndrome after onset of severe aplastic anemia. The donor sibling also found to be developed the acute lymphoblastic leukemia after diagnosing the hypocelluarity of bone marrow. The event of finding these two familial cases shows the bone marrow failure syndrome to be inherited [50].

HAAA has not been clarified with any genetic tendency [18]. Mutation in genes of the telomere repair complex, *TERC *(the gene for the RNA component of telomerase) and *TERT *(the gene for the telomerase reverse transcriptase catalytic enzyme), reduce the marrow regenerative capacity, making genes mutation carriers susceptible to the development of aplastic anemia once it has been started [42].

## Clinical features

Most of the clinical features relating to aplastic anemia following the hepatitis include: Pallor and multiple skin bleeding [11], lymphocytopenia, hypogammaglobulin [51] low number of CD8/T cell ratio [12] and increased number of cytotoxic cells [48] Neutropenia, fever [17]. Bacterial and fungal infection may emerge as secondary in presenting the disease [2]. Later complications may develop especially involving myelodysplasia [4]. The victims of severe aplastic anemia following the hepatitis experience a severe immune deficiency that might be either due to the hepatitis or aplastic anemia that is yet to be discovered [51].

## Diagnosis of hepatitis associated aplastic anemia

On a course of HAAA, hepatitis can be detected on some of the following parameters: subsequent increase in serum Alanine Trasnaminase (ALT), Aspartate Transaminase (AST), by at least three times above the normal values which are 6 to 41U/l, 9-34U/l, 5-58U/L for ALT and AST respectively [12,18,30,35,36,43,52,53], increase in serum Alkaline phosphatase (ALP), gamma glutaryl transferase (GGT) and billirubin (39-117U/l, 5-58 U/l, and 2-7 micromol/L, respectively). Peripheral blood count can be determined by Flow cytometry analysis with directly conjugated monoclonal antibodies for CD2, CD3, CD4, CD8, CD19 and HLA-DR, whereas haematopoietic failure with bone marrow hypocellularity can be elucidated in terms of absolute neutrophil count (less than 500 per mm^3^), Platelet count (less than 20,000 per mm3), Reticulocyte count (less than 60,000 per mm^3^) [53] and Protrombin Index (%): normal value 70-100% [24]. To establish the onset of pancytopenia following hepatitis, hypocelluarity of bone marrow below 50% might be obtained by bone marrow aspiration [14] and trephine biopsy [11].

Various virological and serological markers are available for the detection of hepatitis A, B, C, D, E, G, TTV and parvovirus. Among these tests, anti-HAV Ig total antibodies, HBsAg, HB core antigen, HBsIgG antibodies, various HCV recombinant antigens and hepatitis E virus IgM and IgG are being in use. However, to determine causative nature of all hepatitis viruses, RNA genome of RNA containing viruses such as HCV, HDV, HEV and HGV can be qualitatively detected by RT-PCR reaction and DNA of parvovirus B19 and TTV can be detected by Nested PCR [14,53]. IgG antibody for Cytomegalovirus, EBV and parvovirus has been found a useful tool for the diagnostic purposes [18]. However, serological and virological parameters for hepatitis A, B, and C were found negative in majority of the HAAA cases reported in several studies [17,23,27].

## Treatment

The standard therapy which is employed for the treatment of HAAA is allogenic bone marrow (BM) transplantation treatment from HLA matched siblings [13,54]. HAAA is mostly occurring in children and it would be easier to find the HLA matched donor. As HAAA shows poor prognosis, most often it has been treated by hematopoietic stem cell transplantation [26,55]. Immunosuppressive therapy has proved effective after BM transplantation. Various immunosuppressive drugs named Antithymocyte Globulin (ATG) and Cyclosporine have been administered without eliciting any acute side effects. Steroids such as Glucocorticoids have also been employed in combination with immunosuppressive medications for the treatment of the HAA patients [26]. A durable remission from HAA has been achieved by the administrating high dose of Cyclophosphamide (CY), a highly immunosuppressive, which elicits its effect by readily destroying the lymphocytes and committed myeloid cells. The haemopoietic stem cells are not susceptible to the toxic effects of the CY due to releasing the aldehyde dehydrogenase enzyme which inactivates the drug. However, restoration of haematopoesis process may achieved by high dose of CY which mediates autoimmune attack on haematopoiectic stem cells HSCs [13].

Patients irresponsive to the IST are prone to be cured by unrelated donor bone marrow transplantation [18]. Immunosuppressive therapy might have proved as a safe and alternative treatment for HAAA after of bone marrow or haemotopoietic stem cell transplantation [55]. Several studies showed that Parvovius induced aplastic anemia improves by the administration of retroviral therapy [21,56]. Antiviral therapy for treating hepatitis B associated HAAA has been unknown yet; however it has been tested by administrating the nucleoside analogs, lamiviudine, against the aplastic anemic secondary to hepatitis B virus infection and remission occurs from the severe aplastic anemia accompanied with the hepatitis B viral infection. Interferon, an effective therapy for the hepatitis B and C viral infections, cannot be employed as a potential approach for the HAAA because of its mylosuppressive effects [21]. Acyclovir has been used for the treatment of aplastic anemia caused by the Epstein Bar virus [32]. The blood count comes to the normal range after five years of treatment [55], however, chances of recovery from hepatitis associated acquired aplastic anemia is rare [57].

Growth factors deficiencies have been found to be responsible for the majority of the aplastic anemic cases [1]. As these growth factors released by stromal cells are essential for the survival, proliferation and differentiation of hematopoietic stem cells [58,59], a transient increase in granulocytes has been found effective in most aplastic anaemic trials by administrating the erythropoietin, growth factors, granulocyte colony-stimulating factor, granulocyte macrophage colony-stimulating factor, interleukin-3 [1] and androgens [5].

The limiting factors in success of immunosuppressive therapy are found to be the extent to which organ has been destructed, tissue regeneration capacity and most importantly pharmacology effect of drugs that is not sufficient for uncontrolled potent immune response [1].

The survival of the patients treated with hematopoietic cell transplantation and response rate to immunosuppressive therapy found to be 85% and 70% respectively [7]. Children response better than the adults to bone marrow transplantation and survival rate with Bone marrow transplantation from HLA matched donors is found to be similar as that for the non hepatitis associated aplastic anemia [14].

It has also been reported that parameters of liver dysfunctioning tend to improve when pancytopenia starts presenting itself [18,28]. Although majority of the patients survives after aplastic anaemia tends to have complete recovery, the mortality rate is yet very high [52]. The mean survival rate after developing the severe bone marrow aplasia has been 2 months and fatality rate ranges from 78-88% [28,60,61].

## Conclusion

HAAA is a well documented and diverse variant of clinical syndrome of aplastic anemia, in which an acute attack of hepatitis leads to the marrow failure and pancytopenia that may be acute or chronic. This disorder has been reported in 2-5% cases in West, 4-10% in Far East and high in area of low socioeconomic status. HAAA is not related to age, sex and severity of hepatitis, predominantly it has been found in children, adolesecent boys and in young aged men. A number of hepatitis viruses manifest the disease symptoms. Amongst the hepatitis viruses, HBV, HCV and HGV seropositivity has been mostly commonly observed in reported cases of HAAA. The causative agent of HAAA can be detected using hematological, biochemical, immunological and virological markers. The clinical features relating to HAAA are pallor and multiple skin bleeding, lymphocytopenia, hypogammaglobulin, low number of CD8/T cell ratio and increased number of cytotoxic cells, neutropenia and fever etc. For HAAA, immunosuppressive therapy is more effective after BM transplantation.

## Abbreviations

AA: Aplastic anemia; HAAA: hepatitis associated aplastic anemia; HCV: hepatitis C virus; NANBH: non-A non-B hepatitis; HGV: hepatitis G virus; HBV: hepatitis B virus; HAV: hepatitis A virus; HDV: hepatitis delta virus; HEV: hepatitis E virus; ELISA: enzyme linked immunosorbant assay; PCR: polymerase chain reaction; TTV: transfusion transmitted virus; INF-γ: interferon gamma; TNF-α: tumor necrosis factor alpha;

## Competing interests

The authors declare that they have no competing interests.

## Authors' contributions

BR reviewed the literature, and wrote the manuscript. MI & SARS edited the manuscript. SB, AMB, AH, IR, MA, LA and SB helped BR in literature review. All the authors read and approved the final manuscript.
